# Chemical footprints mediate habitat selection in co-occurring aphids

**DOI:** 10.1093/beheco/arac076

**Published:** 2022-08-20

**Authors:** Mitzy F Porras, Nathaniel McCartney, Günther Raspotnig, Edwin G Rajotte

**Affiliations:** Department of Entomology, Pennsylvania State University, University Park, PA, USA; Department of Entomology, Pennsylvania State University, University Park, PA, USA; Institute of Biology, Karl-Franzens University, Universitaetsplatz, Graz, Austria; Department of Entomology, Pennsylvania State University, University Park, PA, USA

**Keywords:** cuticular compounds, plant microsites, Rhopalosiphum maidis, Rhopalosiphum padi

## Abstract

Habitat selection is a critical process that shapes the spatial distribution of species at local and regional scales. The mechanisms underlying habitat preference rely on environmental factors, species traits, and ecological interactions with other species. Here, we examined spatial segregation between two co-occurring aphid species (*Rhopalosiphum maidis* and *R. padi*) on wheat plants. We hypothesized that spatial segregation between these aphid species was mediated by aphid cuticular compounds left as chemical “footprints” on plant surfaces. Combining field and laboratory experiments, we first examined how plant microsites alter fitness by measuring the fecundity of each species. Next, we tested whether intra- and interspecific pre-inhabitation modified habitat selection in both aphid species. Both aphid species preferred and exhibited higher fecundity on wheat stems versus leaves. Laboratory trials showed that *R. maidis* pre-inhabitation altered *R. padi* spatial preference. By gas chromatography-mass spectrometry analysis and bioassays testing the effects of aphid density and footprint extracts, we found a density-dependent response, with *R. padi* avoiding locations previously inhabited by *R. maidis*. The chemical analysis of footprint crude extracts revealed a highly abundant compound, 1-hexacosanol, and when presented in the synthetic form, also elicited *R. padi* displacement. Altogether, it indicated that *R. maidis* footprints altered *R. padi* habitat selection with cuticular compounds playing a relevant role in the habitat selection process in co-occurring aphid species.

## Teaser

Traits like body size, thermal sensitivity, and pheromone production/response can alter a species’ habitat preferences with implications for the ways animals compete for limited resources. We used field and laboratory experiments to explore how two aphid species compete for the same host plant microsites. We showed that a cuticular chemical signal deposited by the competitor aphid species contributes to spatial segregation between the co-occurring species.

## INTRODUCTION

Habitat selection is a process shaped by multiple factors such as environment, species traits, and ecological interaction with other species (Rosenzweig 1991). Competition among species may reduce settlement in high-quality areas leading to major impacts on fitness (Connell 1961, Hui and Moyse 2018). The mechanisms used to compete for resources may involve anatomical, physiological, or behavioral traits such as wing shape or habitat selection for oviposition (Jaenike 1982, Winkler and Leisler 1985, Holt 1987). Species traits such as chemical signals are another way in which species interact with one another and can affect the way they exploit resources (Regos et al. 2019). Many animals that compete for resources use chemical signals that mediate dispersion, aggregation, or territorial marking (Faulde et al. 1990, Kosaki 1996, Wheeler and Cardé 2014, Barbero 2016). However, there is much to be explored about the role of chemical signals on habitat selection among insect species sharing the same limited resources (van Wilgenburg et al. 2012). Phytophagous insects co-occurring on the same host plant are a good model to examine whether chemical signals influence habitat preference. For example, the aphids *Rhopalosiphum maidis* and *R. padi* are frequently observed on the same wheat plant, but *R. maidis* occurs on the stem while *R. padi* inhabits the leaves(Barro and Wallwork 1992, Harrington et al. 2007, Porras et al. 2020). Both aphid species are hemimetabolous phloem-feeders, overwinter as egg on *Prunnus virginiana* and in spring migrate to grasses, including wheat, barley, where parthenogenetically reproduce (Dixon 1971, Foott 1977) and competition can reduce fitness, and the strength of this outcome can vary with environmental temperature and viral infection (Porras et al. 2020). Chemical signaling may also mediate host plant exploitation with implications for species’ habitat selection (Denno et al. 1995, Inbar and Wool 1995, Smith et al. 2008).

Chemical signals used by animals are typically produced by exocrine glands and exhibit different levels of volatility, aerial or aquatic dispersion, or dynamics on surfaces such as the cuticle of the organism or other surfaces in the habitat. While animals may actively release chemical signals into their environment, they may also passively deposit compounds from their cuticles as they walk on substrates (“footprints”). For example, mammals, reptiles, and arthropods, including crustaceans, spiders, and insects, use footprints to mark resources, thus affecting intra and interspecific habitat selection (Kosaki 1996, David 2000, Le Goff et al. 2009, Lecchini et al. 2010, Mestre et al. 2014, Guillem et al. 2016, Heyman et al. 2017). Aphids may use chemical footprints in habitat selection. Although a previous study suggested quantitative cuticular hydrocarbon differences among *Rhopalosiphum* species (Lazzari et al. 1991), the collection method (complete body washes) did not allow for quantitative or qualitative comparisons of residues deposited by the species on substrates and may even have included extracts from internal tissues such as compounds from the hemolymph. Using other protocols such as aphid footprint analysis washes may allow a more precise measure of the chemical signal and provide a better understanding of the role of chemical signals on the competition between species that share the same limiting resources.

Here, we investigated whether habitat choices (i.e., stem or leaf microsites) in co-occurring aphids, *R. padi* and *R. maidis,* were mediated by chemical signaling. We assessed aphid spatial preference when species were alone or together on the same plant as well as fecundity, measured as per-capita offspring, for both stem and leaf. We then evaluated whether intra and/or interspecific pre-inhabitation by either species altered each species’ spatial preference using choice test arenas. Next, we measured the effects of *R. maidis* density on *R. padi* spatial preference. Finally, we identified the major compounds in the cuticular footprints of *R. maidis* using gas chromatography-mass spectrometry (GC-MS) and tested whether the dominant compound in *R. maidis* cuticular footprints modified *R. padi* spatial preference. Overall, our results indicated that these aphid species compete for space, and *R. maidis* footprints alter *R. padi* habitat choice. Thus, we suggest that the co-occurrence of aphids on wheat plants can be mediated by interspecific chemical signaling.

## MATERIALS AND METHODS

### Aphid colonies

We obtained our stock of *R. padi* and *R. maidis* colonies from the laboratory of Virology and Nematology at Cornell University and the University of Idaho. The colonies were kept in laboratory conditions, at 20 ± 2 °C, ambient photoperiod on barley (*Horedum vulgaris* L.) and wheat (*Triticum aestivum* L.). Every 2 weeks, new parthenogenetic colonies were started using first instar nymphs; thus, experimental aphids were at the same developmental stage.

### Experiment 1: Aphid preferences for plant microsites

To investigate whether *R. padi and R. maidis* compete for host plant microsites (i.e., stem or leaf), we conducted a field experiment using a raised bed system with natural soil (120 cm × 100 cm × 40 cm, 50 cm above ground) at the Pennsylvania State University Horticultural Facility (University Park, PA). Wheat seeds were planted at a density of 1 seed/4.5 cm^2^. We used a paired design, similar to Porras et al. (2020), to examine the effect of interspecific competition on aphid location on the host plant (stems vs. leaves). Experimental factors (independent variables) were the following: 1) no interspecific competition (a single species on a host plant), and 2) interspecific competition (both species on the same host plant). These treatments were evaluated for each aphid species and were replicated six times (*n* = 6) on the same day. The trials consisted of six individuals of a species placed on a plant for no interspecific competition and six individuals of each species simultaneously placed on a plant for interspecific competition (*R. padi*: *n* = 6, and *R. maidis*: *n* = 6, on each of six plants). Apterous aphids were collected from stock colonies (grown on *H. vulgaris*) and transferred to petri dishes using a paintbrush. Aphids resided inside the petri dishes on wheat leaves for 4 h. We then transferred six adults of each species to a 5-week-old plant, placing the aphids on the stem 6 cm above the soil surface (no competition). For the interspecific competition, we transferred six individuals of each species (one aphid at a time, alternating between species) at the same plant height (6 cm from the soil surface). Then, plants were enclosed in a transparent acrylic tube (4.5 cm D × 35 cm L) that had two windows (3 cm × 5 cm) covered with Lumite fabric (OHCO, Georgia, GA). After 24 h, we counted the number of individuals of each species on the stem (0–15 cm above the soil surface) and on the leaves.

### Experiment 2: Effects of plant microsite on aphid fecundity

To examine whether host plant microsites affect *R. padi* and *R. maidis* fecundity, we recorded the per-capita offspring of each species on the stems and leaves under greenhouse conditions. The trial consisted of one aphid per plant. Each treatment was replicated 15 times (*n* = 15). We placed a 3-day-old aphid adult (from the *H. vulgaris* colonies) inside a leaf cage made from mesh-topped foam rings placed on each side of the leaf and held in place by three splayed staples that were inserted in each ring, clamping them snugly against the leaf (dimensions 36.5 × 25.4 × 9.5 mm) (Bioquip Products, Inc.). Cages were attached to a 4-week-old wheat plant (cv. Spring) on either a stem or a leaf. The aphid was allowed to reproduce overnight. The following morning, we removed the adult and all but one first-instar nymph from the plant microsite inside the leaf cage. Once the nymph reached reproductive age (usually 7 days), we recorded and removed the offspring every day until dead.

### Experiment 3: Effect of intra and interspecific pre-inhabitation on spatial preference of aphids

We used a paired design to evaluate the effect of pre-inhabitation (no and yes) on the spatial preference of aphids. Our treatments to evaluate the impacts of intraspecific pre-inhabitation were the following: *R. padi* on 1) uninhabited tubes (control) and 2) pre-inhabited by conspecifics; *R. maidis* on 1) uninhabited tubes (control) and 2) pre-inhabited by the conspecifics. To examine interspecific pre-inhabitation, the treatments were the following: *R. padi* on 1) uninhabited tubes (control) and 2) pre-inhabited by *R. maidis*; *R. maidis* on 1) uninhabited tubes (control) and 2) pre-inhabited by *R. padi*. Each treatment was replicated 20 times.

The experimental unit consisted of adult aphids placed into a dual-choice arena consisting of two joined plastic tubes (3 cm D × 5 cm L) where one tube was pre-inhabited and the other clean (uninhabited). To produce the pre-inhabited tube, we placed 20 adult aphids in the inner surface of the tube using a paintbrush, then covered each end using a plastic lid with hole (2 cm diameter for air flow), covered by Lumite fabric (OHCO, Georgia, GA) and removed the aphids after 24 h. We then attached a clean tube to the end of the pre-inhabited tube using Parafilm. Next, we placed the joined tubes (experimental arena) on a cold plate to ensure aphids are evenly distributed in the connection point of the tubes, and released 20 chilled (5 °C) *R. padi* adults by inserting a single aphid at a time using a paintbrush from alternating ends of the arena (Supplementary Figure 1). Finally, after 6 h, we recorded the number of aphids in the pre-inhabited and clean tubes. All our choice test experiments were conducted in the laboratory at 23 ± 2 °C and 60% RH.

### Experiment 4: Effects of aphid density on habitat selection and chemical analysis of footprints

#### Live aphid bioassay

Because *R. padi* avoided pre-inhabited microsites by *R. maidis*, we examined whether the *R. padi* response was modulated by *R. maidis* density. We then used a factorial design to measure the effects of *R. maidis* density on *R. padi* spatial preference (number of individuals) in a choice test arena. Four densities of *R. maidis* (2, 6, 10, and 20 adults) were allowed to occupy one tube for 24 h and removed as described in the previous experiment. Next, we joined this pre-inhabited tube to a clean tube forming the choice arena. We released 20 *R. padi* adults in the joint (Supplementary Figure 1) and after 6 h recorded the number of aphids on each side of the arena (pre-inhabited or non-inhabited). A pair of joined non-inhabited tubes was used as a control. Each *R. maidis* density was replicated ten times (*n* = 10).

#### Bioactivity of footprint extracts

To examine whether the *R. padi* spatial preference is mediated by a *R. maidis* density-dependent chemical signal, we extracted *R. maidis* footprints by placing 2, 6, 10, and 20 adults inside glass vials (2.5 cm D × 9.5 cm L, previously washed twice using 5 mL of hexane, wrapped in aluminum foil and dried overnight at 60 °C) and removed the aphids after 24 h. Chemical footprints from a vial’s inner surface were extracted by washing the *R. maidis* footprints deposited on the glass surfaces with *n*-hexane (1000 µL). The *n*-hexane was pulled from the vial using a glass syringe, transferred to a 4 mL glass vial and evaporated under nitrogen to 150 µL, transferred to a glass conical insert, evaporated again to approximately 100 µL, and stored at −80 °C. The activity of footprint extracts for each *R. maidis* density was tested by evenly spreading each extract on the inner surface of a clean glass tube using a glass syringe and dried at room temperature 23 ± 2 °C. We then joined the treated tube to a clean tube. Next, we released 20 *R. padi* adults into the tube at the joint as described above (*n* = 3 replicates per treatment).

#### Identification and quantification of footprint components

To identify and quantify the components of the cuticular compounds in the footprints of *R. miaidis*, we prepared extracts from five aphid densities (0, 25, 50, 100, and 300) as described above by placing the aphids in glass vials (50 aphids per vial to avoid alarm pheromone release), left them inside the vial for 24 h and washed the inner surface of the vial with *n*-hexanes. Solvent washes were pulled using a glass syringe and evaporated as previously described. The samples were analyzed by gas chromatography-mass spectroscopy (GC-MS) in both electron impact (EI) and chemical ionization (CI) modes using an Agilent Technologies (Little Falls, DE) 6890 gas chromatograph fitted with an HP-5MS bonded phase capillary column (0.25 mm × 0.25 µm × 30 m; Agilent Technologies) interfaced to an Agilent 5973 mass spectrometer. The column temperature was programmed from an initial temperature of 50 °C, with a 1 min hold time, 20 °C min^-1^ to 200 °C, and 4 °C min^-1^ to 300 °C with a 20-min hold at 300 °C. Splitless injections of 1 µL were made with the inlet at 280 °C with a split time of 0.75 min and helium carrier gas flow rate of 1.0 mLmin^-1^. EI analysis used the default settings (ion source: 230 °C, quadrupole: 150 °C, and spectra generated at 70eV). CI analysis was performed in scan mode using a source temperature of 250 °C, a quadrupole of 150 °C, an emission of 150 µA, an ionization energy of 60eV, and an isobutane reagent gas flow of 14%. Identification of analytes was performed using the NIST 17 library, published retention index values, CI spectra, and authentic standards. Components of the aphid footprint extracts were identified and quantified by external standard calibration of total ion chromatogram peak areas using an authentic standard of 1-hexacosanol and tetracosane (Sigma-Aldrich, St. Louis, MO). To account for substantial differences in instrument responses to alcohols relative to alkanes, alkenes, and aldehydes, alcohols were quantified using 1-hexacosanol, while all other compounds were quantified using tetracosane. Fourteen standard dilutions containing tetracosane and 1-hexacosanol in equal proportions were analyzed, covering a range from 40 pg to 300 ng per µL. Total ion current (TIC) peak areas and standard concentrations were log-transformed to correct for heteroscedasticity and polynomial trend-lines were generated for both standards (*R*^2^ = 0.9997 and 0.9995, respectively). The regression equations were used to calculate concentrations of analytes in each sample, which multiplied by sample volume gave the total masses of analytes in the extract. Per-aphid contributions to these masses were calculated by dividing the analyte mass by the number of aphids used to generate footprint residues for each extract. Single-point standard addition quantification was performed on selected extracts to verify that no significant sample matrix effect interfered with the quantification of naturally produced 1-haxacosanol.

#### Bioactivity of synthetic major footprint compound

To examine whether the most abundant compound in *R. maidis* cuticular footprints alters *R. padi* spatial preference, we evaluated five quantities of synthetic 1-hexacosanol that corresponded to the number of adults used in the live aphid bioassay. These quantitates were chosen to conservatively approximate the quantities deposited in footprints over a 24 h for different numbers of individuals based on observed per-aphid contributions of about 20 ng (0 adults = 0 ng, 2 adults = 40 ng, 6 adults = 120 ng, 10 adults = 200 ng, and 20 adults = 400 ng) calculated using the regression described above. These quantities were chosen to conservatively approximate the quantities deposited in footprints over a 24 h for different numbers of individuals based on observed per-aphid contributions of about 20 ng (TCI America, purity 95%) to a glass tube, allowed the solvent (*n*-hexane) to evaporate and then joined a clean glass tube to the end of the treated tube using Parafilm. Next, we released 20 *R. padi* adults at the joint, and after 6 h, the distribution was recorded (*n* = 3 replicates per treatment).

### Statistical analysis

Data from all the experiments were tested for normality (Shapiro-Wilks) and homogeneity of variance (Levene’s test) assumptions. In experiment 1, to identify the effect of interspecific competition on the spatial distribution of aphid species, we conducted an analysis of variance with two factors (two-way analysis of variance [ANOVA], interspecific competition and plant microsite location (leaf, stem), and the interaction of both factors. Data from experiment 2 were analyzed using a two-way ANOVA, where the fecundity was compared between the species and the host plant microsite location and their interaction.

In experiment 3, we used a generalized linear model (GLM) with a binomial distribution using a likelihood test to test the effect of intra and interspecific pre-inhabitation on aphid spatial preference. For experiment 4, we compared *R. padi* spatial preference among areas pre-inhabited by different *R. maidis* densities using a GLM with a binomial distribution. Next, we compared the bioactivity of *R. maidis* footprint extracts from different densities and the synthetic form of the major *R. maidis* footprint extract on *R. padi* spatial preference following the same statistical procedure (GLM with a binomial distribution). All data were analyzed using the R programming environment (v. 3.4.3., CRAN project) (Team 2013).

## Results

### Experiment 1: Aphid preferences for plant microsites

**Figure 1:**
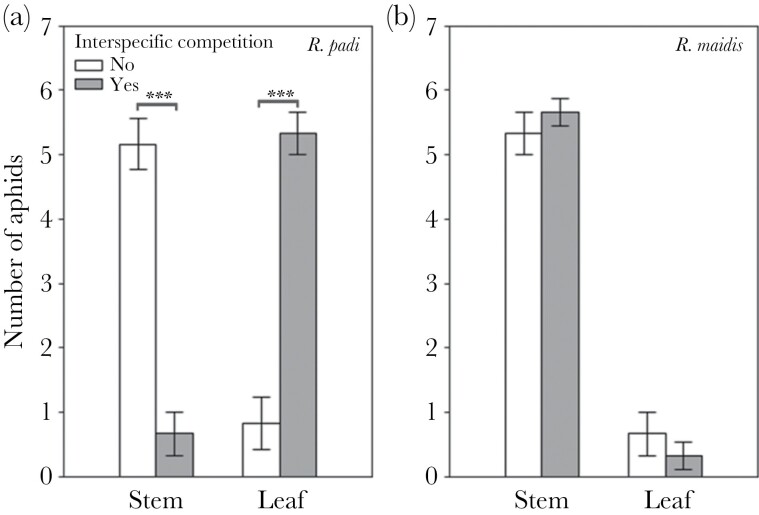
Plant microsite preference of aphids (stems vs. leaves) with and without interspecific competition. *Rhopalosiphum maidis* altered *R. padi* settlement on the host plant. Plant microsite preference of (a) *R. padi* and (b) *R. maidis*. Bars, mean ± SE, *n* = 6. Significance level is ***P < 0.0001.

In the raised-bed system in the field where aphids could freely choose plant microsites, both aphid species, when alone were found on the stem. However, when both species were present, *R. maidis* significantly reduced the number of *R. padi* on both stems and leaves (two-way ANOVA: *F*_3,20_ = 40.55, *P* < 0.0001; Figure 1b; Supplementary Table 1). The number of *R. maidis* was significantly higher on the stem, and its plant microsite preference was not affected by *R. padi* (two-way ANOVA: *F*_3,20_ = 107.61, *P* < 0.0001; Figure 1a; Supplementary Table 1).

### Experiment 2: Effects of plant microsite on aphid fecundity

**Figure 2:**
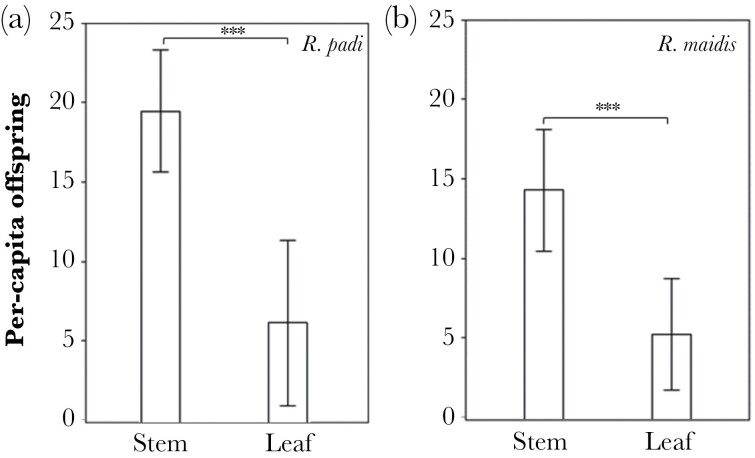
Effect of host plant microsite on the aphid per-capita offspring. The fecundity of both species was higher on the stem than the leaf. (a) *Rhopalosphiphum padi* and (b) *R. maidis*. Bars, mean ± SE, *n* = 15, significance level is ***P < 0.001.

Per-capita offspring of both species, when alone, was significantly higher on the stem compared to the leaf (two-way ANOVA: *F*_3,56_ = 100.66, *P* < 0.0001; Supplementary Table 2; Figure 2). The number of *R. padi* offspring was four times higher on the stem than on the leaf (Figure 1a), *R. maidis* offspring followed a similar trend as *R. padi* (Figure 2b).

### Experiment 3: Effect of inter and intraspecific pre-inhabitation on the spatial preference of aphids

**Figure 3:**
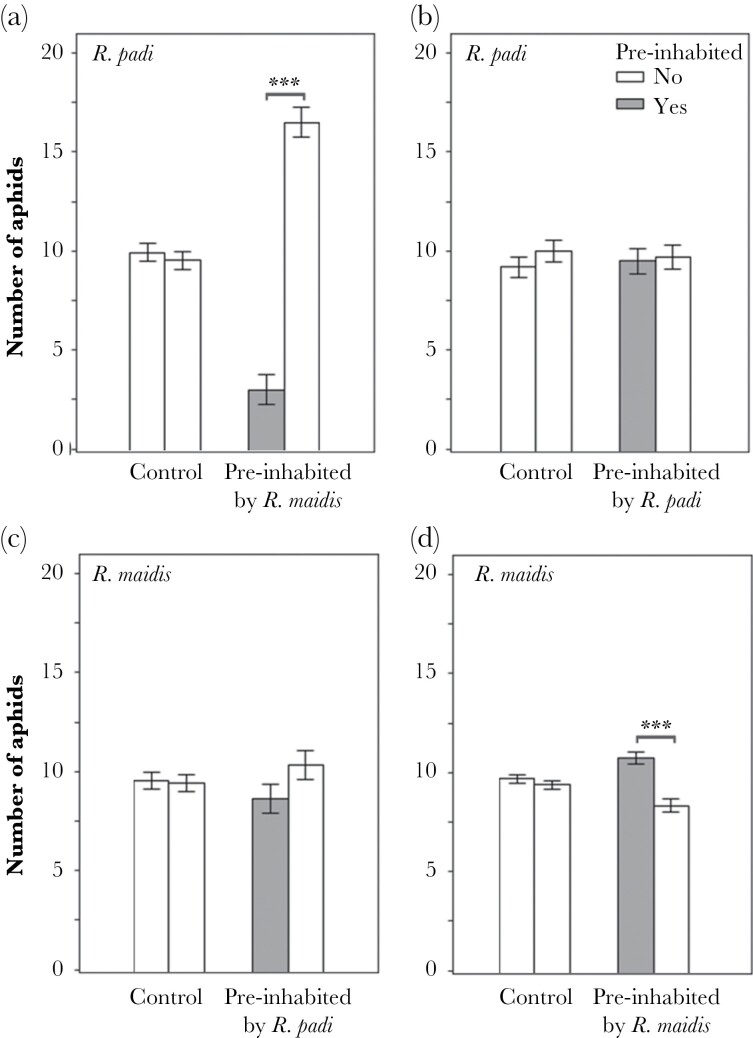
Effect of inter and intraspecific species pre-inhabitation. *Rhopalosiphum padi* avoids micosites pre-inhabited by *R. maidis*. (a) *R. padi* preference on pre-inhabited microsites by *R. maidis*, (b) *R. maidis* preference on pre-inhabited microsites by *R. padi*. (c) Effect of intraspecific pre-inhabitation by *R. padi*, (d) effect of intraspecific pre-inhabitation by *R. maidis.* Bars, mean ± SE (*n* = 20), significance level is ***P < 0.001.

Habitat preference of *R. padi* was affected by *R. maidis* pre-inhabitation (χ^2^ = 128.69, df = 3, *P* <0.0001; Table 1; Figure 3a), while pre-inhabitation by *R. padi* did not affect the *R. maidis* preference (*P =* 0.69; Figure 3b) in the experimental arena. Trials testing the effects of conspecific pre-inhabitation showed that *R. padi* pre-inhabitation did not affect the spatial preference of its conspecifics (*P* = 0.104; Figure 3c), while *R. maidis* preferred microsites pre-inhabited by its conspecifics (χ^2^ = 12.15, df = 3, *P* = 0.0007; Table 1; Figure 3d).

**Table 1:** Summary of parameters of generalized linear mixed models with binomial distribution fitting responses of individual proportion of aphids as a function of the treatment of pre-inhabitation or no-inhabitation by conspecific or heretospecific of aphids *R. maidis* and *R. padi*

Model effect	Β	SE	z	*P*
Heterospecific *R. maidis* pre-inhabitation effect on *R. padi*				
Intercept	0.050	0.100	0.500	0.617
Treatment *R. maidis*	−0.885	0.148	−5.989	<0.001
No-inhabited microsite pre-inhabited microsite	−0.090	0.141	−0.636	0.525
Treatment *R. maidis* × No-inhabited microsite pre-inhabited microsite	1.761	0.209	8.423	<0.001
Cospecific *R. maidis* pre-inhabitation effect on *R. maidis*				
Intercept	0.145	0.071	2.049	0.040
No-inhabited microsite pre-inhabited microsite	−0.295	0.100	−2.947	0.003

### Experiment 4: Effect of aphid density on habitat selection and chemical analysis of footprints

**Figure 4:**
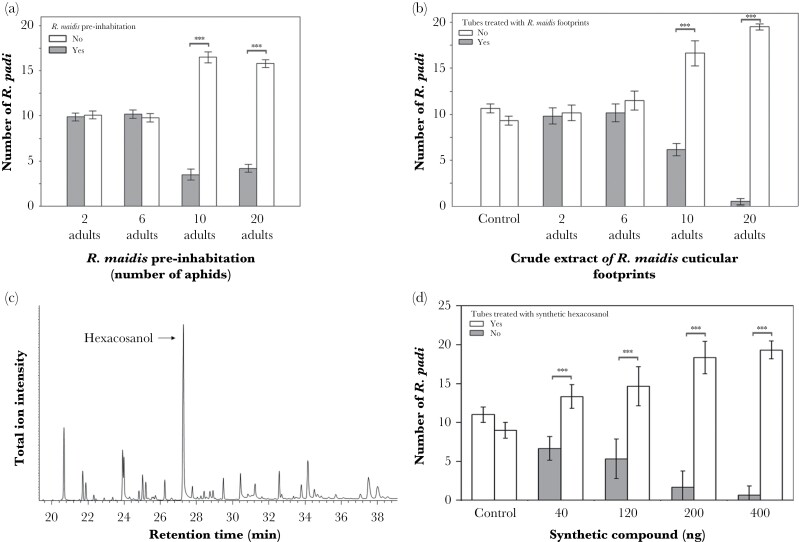
Spatial preference of *Rhopalosiphum padi* in response to *R. maidis* footprint extracts. *R. padi* avoided microsites pre-inhabited by *R. maidis*. Effects of *R. maidis* density (2, 6, 10, and 20) on the spatial preference of (a) *R. padi* (*n* = 10), (b) crude extracts (*n* = 3), (c) GC-MS total ion chromatogram of the *R. maidis* cuticular footprints, (d) Response of *R. padi* to synthetic 1-hexacosanol (*n* = 3) (mean ± SE). Significance level is ***P < 0.0001.

The *R. padi* spatial preference was significantly affected by higher *R. maidis* density (Table 2). Differences in *R. padi* preference were observed when the individuals were exposed to experimental arenas with *R. maidis* pre-inhabited microsites with ten and 20 adults (Figure 4a, Supplementary Table S2). The same trend was observed in bioassays testing footprint extract activity, *R. padi* significantly avoided treated microsites with extracts from ten and 20 *R. maidis* adults (χ^2^ = 135.38, df = 9, *P* < 0.0001; Table 3; Figure 4b; Supplementary Table S4).

**Table 2:** Parameter summary of generalized linear mixed models with binomial distributions of *R. padi* as functions of *R. maidis* density

Model effect	Β	SE	z	*P*
Density of *R. maidis*				
Intercept	−1.531	0.195	−7.856	**<0.001**
Treatment 2 adults	1.084	0.243	4.463	**<0.001**
Treatment 20 adults	0.173	0.257	0.675	0.500
Treatment 6 adults	1.371	0.241	5.687	**<0.001**
Treatment Control	1.621	0.219	7.399	**<0.001**
Non-inhabited microsite pre-inhabited microsite	3.082	0.269	11.436	**<0.001**
Treatment 2 adults × Non-inhabited microsite pre-inhabited microsite	−2.187	0.339	−6.460	**<0.001**
Treatment 20 adults × Non-inhabited microsite pre-inhabited microsite	−0.369	0.362	−1.017	0.309
Treatment 6 adults × Non-inhabited microsite pre-inhabited microsite	−3.042	0.336	−9.055	**<0.001**
Treatment Control × Non-inhabited microsite pre-inhabited microsite	−3.262	0.304	−10.716	**<0.001**

**Table 3:** Parameter summary of generalized linear mixed models with binomial distributions of *R. padi* as functions of crude *R. maidis* cuticular footprints extracts

Model effect	Β	SE	z	*P*
Crude extract of *R. maidis* cuticular footprints				
Intercept	0.619	0.271	2.287	**0.022**
Treatment 2 adults	−0.351	0.376	−0.934	0.350
Treatment 20 adults	2.020	0.584	3.459	**<0.001**
Treatment 6 adults	−0.283	0.377	−0.750	0.453
Treatment Control	−0.485	0.374	−1.297	0.195
Clean microsite treated microsite	−1.238	0.383	−3.234	**0.001**
Treatment 2 adults × Clean microsite treated microsite	0.564	0.533	1.059	0.289
Treatment 20 adults × Clean/treated microsite	−4.040	0.826	−4.891	**<0.001**
Treatment 6 adults × Clean microsite treated microsite	0.565	0.533	1.061	0.289
Treatment Control × Clean microsite treated microsite	1.038	0.529	1.961	**0.049**

GC-MS analysis detected a major compound comprising approximately 34% of the total extract that was identified as n-hexacosanol by matching retention time as well as EI and CI spectra to an authentic standard (Figure 4c). The per-insect contribution of this compound was estimated as 24.3 ng per *R. maidis* adult. Other compounds were found in much lower abundance such as 1-octacosanol (7.8%), 1-tetracosanol (6.8%), pentacosane (3.5%), heptacosane (2.1%), hexacosanal (1.9%), octacosanal (0.9%), and triacontanal (0.6%). Bioassays testing synthetic 1-hexacosanol in quantities approximating those observed for the major compound in the footprints showed that the *R. padi* spatial preference was significantly reduced in microsites treated with any of the 1-hexacosanol quantities (Figure 4d; Table 4; Supplementary Table S4).

**Table 4:** Parameter summary of generalized linear mixed models with binomial distributions fitting responses of proportion of individuals of *R. padi* as functions of synthetic 1-hexacosanol

Model effect	Β	SE	z	*P*
Synthetic hexacosanol				
Intercept	1.011	0.292	3.465	**<0.001**
Treatment 200 ng	1.386	0.551	2.517	**0.012**
Treatment 40 ng	−0.3185	0.400	−0.796	0.426
Treatment 400 ng	2.356	0.776	3.035	**0.002**
Treatment Control	−1.212	0.391	−3.104	**0.002**
Clean microsite treated microsite	−2.023	0.413	−4.900	**<0.001**
Treatment 200 ng × Clean microsite treated microsite	−2.773	0.779	−3.559	**<0.001**
Treatment 40 ng × Clean microsite treated microsite	0.637	0.566	1.125	0.260
Treatment 400 ng × Clean microsite treated microsite	−4.711	1.098	−4.292	**<0.001**
Treatment Control × Clean microsite treated microsite	2.424	0.552	4.389	**<0.001**

## DISCUSSION

Our field and laboratory trials showed that *R. padi* significantly avoided microsites pre-inhabited by *R. madis*, and the host plant microsites strongly affected fecundity in both species. Our results align with previous studies that showed that *R. padi* spatial preference was significantly affected by *R. maidis* co-occurrence on the same host plant (Porras et al. 2020). However, our results indicate that *R. padi* plant microsite choice is also mediated by an *R. maidis* chemical signal suggesting that *R. maidis* is a more successful species in colonizing the habitat than *R. padi* and thus can outcompete *R. padi*. We showed that chemical signals were involved in the complex suite of mechanisms that explain the co-occurrence of these congeneric aphid species, which includes plant microclimate, aphid thermal tolerance (Porras et al. 2020), and order of species assembly (Barro and Wallwork 1992, Harrington et al. 2007, Porras et al. 2018).

Both aphid species produced the most offspring on the stem, which could be due to higher photosynthate in the stem during wheat development (Thorne 1982, Patrick and Wardlaw 1984). Differences in nutritional content in different plant parts may alter aphid habitat preference with direct impacts on fitness (Awmack and Leather 2002). For example, moths have higher fecundity when feeding on fruits rather than shoots (Du et al. 2015). Similar patterns are exhibited by beetles (Tremmel and Müller 2013), crustaceans (Sterner and Schwalbach 2001), gastropods (Toth and Pavia 2002), and moose (Shipley et al. 1998). Our results suggest that the stem is a more suitable habitat for both aphid species than the leaf; however, the number of *R. padi* offspring was higher than *R. maidis* on the leaves, possibly as an adaptive response enabling it to rapidly colonize a new plant.

Laboratory behavioral bioassays indicated that *R. maidis* co-occurrence and pre-inhabitation significantly reduced *R. padi* settlement in the microsite, as 75% of *R. padi* avoided microsites pre-inhabited by *R. maidis,* suggesting the presence of chemical signals that modified *R. padi* spatial preference. We also found that 62.5% of *R. maidis* preferred microsites pre-inhabited by conspecifics. However, during field trials, we observed that this response was not significant and varied between stem and leaves.

Pre-inhabited microsites and footprint extracts from 10 and 20 *R. maidis* adults elicited *R. padi* spatial preference, which indicates that chemical cues that induced this behavior were successfully extracted into the solvent washes. Gas chromatographic analysis of the *R. maidis* cuticular compounds extracts showed that a major compound, 1-hexacosanol, appears to mediate habitat selection of *R. padi*. Our results align with previous studies that report the repellent activity of hexacosanol in mosquitoes and beetles (Acheuk et al. 2017, Gade et al. 2017). However, other studies have suggested its role as a feeding stimulant in silkworm larvae (Mori 1982). Cuticular compounds can serve as scent markers in arthropods (Blomquist and Bagnères 2010), and because of their chemical stability and low volatility, these chemical signals may remain in the habitat for long periods, facilitating recognition of individuals of the same species as well as territorial marking as observed in ticks, ants, spiders, and snakes (Rechav et al. 1978, Shine et al. 2005, Mestre et al. 2014).

Our GC-MS results indicated additional compounds from the ones found in previous studies (Lazzari et al. 1991), the most prevalent being 1-hexacosanol. Bioassays testing *R. padi* responses to *R. maidis* footprints extract and synthetic 1-hexacosanol showed that *R. padi* avoided microsites with equivalent hexacosanol of two aphid individuals, but in the bioassays with the pre-inhabited microsites and footprint extracts, it took a population of ten individuals before significant differences in *R. padi* spatial choices occurred. If hexacosanol is the main driver of *R. padi* spatial preference, we would expect to observe preference for the uninhabited area including the trials testing two and six adult cuticular extracts of *R. maidis*. However, closely related alcohols such as tetracosanol and octacosanol, alkanes (pentacosane and heptacosane), as well as aldehydes (hexacosanal, octacosanal, and triacontanal) were present at lower concentrations, which possibly affected *R. padi* response. Our work shows the role that cuticular compounds play in the spatial preferences of aphid species and the impacts on fitness. This study is a contribution to our understanding of the mechanisms shaping habitat selection in co-occurring species that share limited resources.

## Supplementary Material

arac076_suppl_Supplementary_Figure

arac076_suppl_Supplementary_Figure_Legend

arac076_suppl_Supplementary_Tables

## Data Availability

Analyses reported in this article can be reproduced using data provided by Porras et al. 2022.

## Notes

We thank T. Carlo, J. Millar, and P. Araujo for their suggestions and guidance on experiments. We also thank N. Bosque-Perez, S. Hermman, S. Gray, and D. Smith for providing aphids, J. Ali for generously providing access to equipment required for this work; S. Diloretto for his continuous support in field experiments. We thank anonymous reviewers for their suggestions and comments. M.F.P. was partially supported by Scholarship Fulbright-COLCIENCIAS and the Entomological Society of America.
