# Supplementary figures and images for: Chemical footprints mediate habitat selection in co-occurring aphids
Source: Behav Ecol. 2022 Aug 20;33(6):1107–14. doi: 10.1093/beheco/arac076 (PMC9735235; doi:10.1093/beheco/arac076)

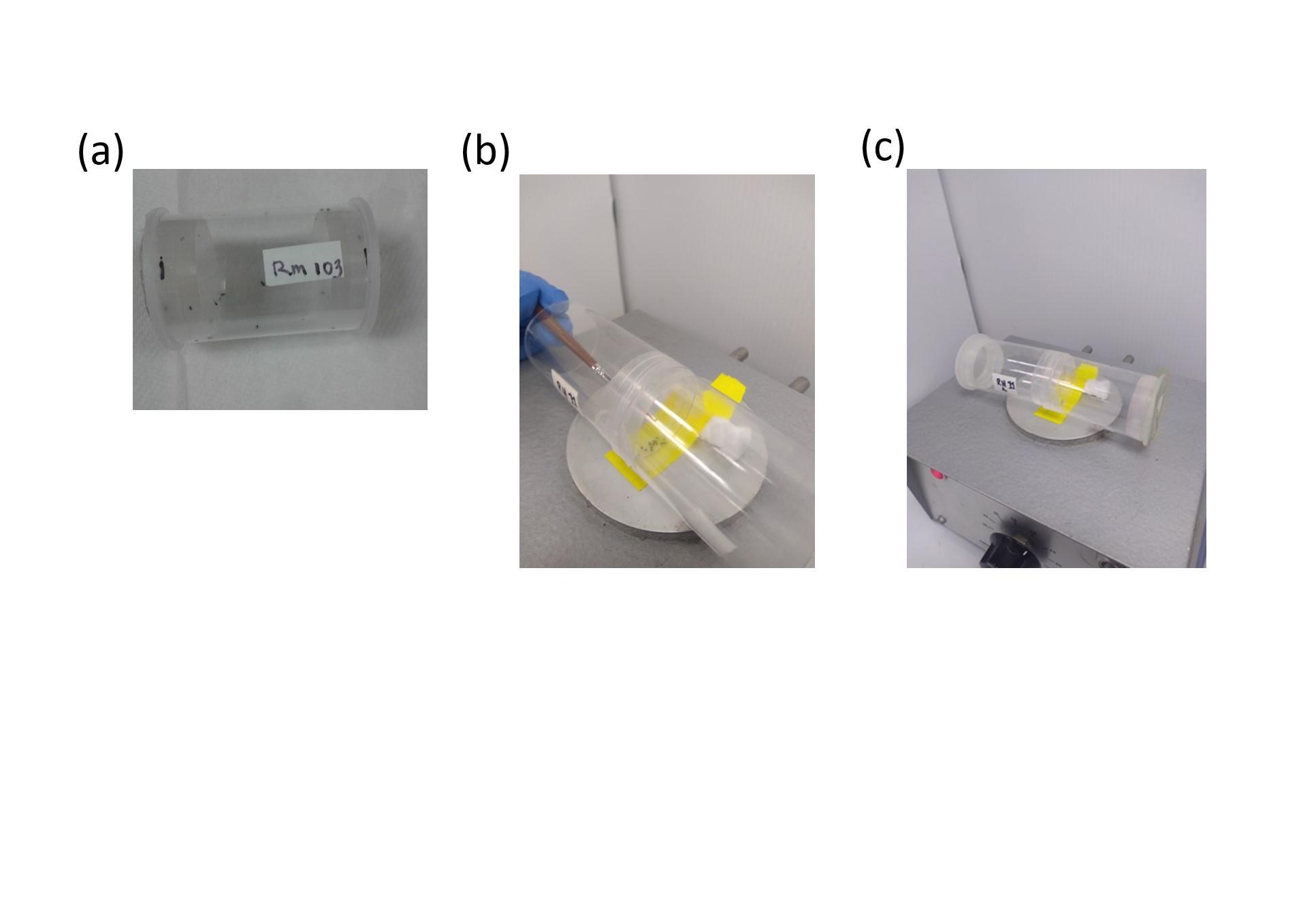

Supplement: arac076_suppl_Supplementary_Figure [file arac076_suppl_supplementary_figure.jpeg]
