# Supplementary material for: Chemical footprints mediate habitat selection in co-occurring aphids
Source: Behav Ecol. 2022 Aug 20;33(6):1107–14. doi: 10.1093/beheco/arac076 (PMC9735235; doi:10.1093/beheco/arac076)
Supplement: arac076_suppl_Supplementary_Figure_Legend [file arac076_suppl_supplementary_figure_legend.docx]

Supplementary Material

**Figure S1**. Choice test arena. (a) Pre-inhabited tube by *R. maidis*, (b-c) pre-inhabited tube and non-inhabited tube on a cold plate.
