# Supplementary material for: Chemical footprints mediate habitat selection in co-occurring aphids
Source: Behav Ecol. 2022 Aug 20;33(6):1107–14. doi: 10.1093/beheco/arac076 (PMC9735235; doi:10.1093/beheco/arac076)
Supplement: arac076_suppl_Supplementary_Tables [file arac076_suppl_supplementary_tables.docx]

Supplementary Material 2

**Table S1.** Parameter estimates of heterospecific presence on aphid spatial distribution

| A) Response of *Rhopalosiphum padi* | | | | | | | | | |
| --- | --- | --- | --- | --- | --- | --- | --- | --- | --- |
| **Term** | | **Estimate** | | **Std Error** | | ***F* Ratio** | | ***P*\|** | |
| Intercept | | 3 | | 0.184466 | | 16.26 | | <.0001* | |
| Location host plant | | 0.08 | | 0.184466 | | 0.45 | | 0.6563 | |
| Competition | | 0 | | 0.184466 | | 0.00 | | 1.0000 | |
| Competition:location plant | | -2.25 | | 0.184466 | | -12.20 | | <.0001* | |
| B) Response of *R. maidis* | |  | |  | |  | |  | |
| Intercept | | 3 | | 0.139443 | | 21.51 | | <.0001* | |
| Competition | | 0 | | 0.139443 | | 0.00 | | 1.0000 | |
| Location host plant | | -2.5 | | 0.139443 | | -17.93 | | <.0001* | |
| Competition:location plant | | 0.1666667 | | 0.139443 | | 1.20 | | 0.2460 | |

**Table S2.** Parameter estimates of the effect of plant microsite on the fecundity of *Rhopalosiphum padi* and *R. maidis*.

| **Term** | **Estimate** | **Std Error** | ***F* Ratio** | **Prob>\|t\|** |
| --- | --- | --- | --- | --- |
| Intercept | 12.48 | 0.39 | 31.56 | <.0001* |
| Specie [*R. maidis*] | -2.91 | 0.39 | -7.37 | <.0001* |
| Plant microsite[Leaf] | -6.21 | 0.39 | -15.72 | <.0001* |
| Specie [*R. maidis*]*Plant microsite [Leaf] | 0.31 | 0.39 | 0.80 | 0.4268 |

Table 2. Model selection by likelihood ratio test fitting responses of the proportion of aphids as a function of the treatment of pre-inhabitation or not of conspecific or heterospecific of the aphids *R. maidis* and *R. padi*, and area pre-inhabited by aphids or not. The best model to explain the proportion is underlined.

| **Model under test** | **χ²** | **df** | **p** |
| --- | --- | --- | --- |
| **Heterospecific *R. maidis* pre-inhabitation effect on *R. padi*** |  |  |  |
| Treatment + area + treatment x area | 128.69 | 3 | < 0.001 |
| Treatment + area | 72.864 | 1 | < 0.001 |
|  |  |  |  |
| **Cospecific *R. padi* pre-inhabitation effect on *R. padi*** |  |  |  |
| Treatment + area + treatment x area | 6.155 | 3 | 0.104 |
|  |  |  |  |
| **Heterospecific *R. padi* pre-inhabitation effect on *R. maidis*** |  |  |  |
| Treatment + area + treatment x area | 1.460 | 3 | 0.691 |
|  |  |  |  |
| **Cospecific *R. maidis* pre-inhabitation effect on *R. maidis*** |  |  |  |
| Treatment + area + treatment x area | 12.155 | 3 | 0.007 |
| Treatment + area | 3.442 | 1 | 0.063 |
| Treatment | 8.720 | 1 | 0.003 |
| Area | 0.002 | 1 | 0.96 |

Table 3. Model selection by likelihood ratio test fitting responses of the proportion of *R. padi* as functions of density of the heterospecific *R. maidis*, crude extract of *R. maidis* cuticular footprints and synthetic hexacosanol, and area pre-inhabited by aphids or not. The best model to explain the proportion is underlined.

| **Model under test** | **χ²** | **df** | **p** |
| --- | --- | --- | --- |
| **Density of *R. maidis*** |  |  |  |
| Treatment + area + treatment x area | 352.1 | 9 | < 0.001 |
| Treatment + area | 222.76 | 4 | < 0.001 |
|  |  |  |  |
| **Crude extract of *R. maidis* cuticular footprints** |  |  |  |
| Treatment + area + treatment x area | 125.7 | 9 | < 0.001 |
| Treatment + area | 66.903 | 4 | < 0.001 |
|  |  |  |  |
| **Synthetic hexacosanol** |  |  |  |
| Treatment + area + treatment x area | 270.76 | 9 | < 0.001 |
| Treatment + area | 118.15 | 4 | < 0.001 |
